# The stability of baseline‐defined categories of alcohol consumption during the adult life‐course: a 28‐year prospective cohort study

**DOI:** 10.1111/add.13949

**Published:** 2017-08-24

**Authors:** Craig S. Knott, Steven Bell, Annie Britton

**Affiliations:** ^1^ Research Department of Epidemiology and Public Health University College London London UK; ^2^ MRC Epidemiology Unit and Centre for Diet and Activity Research (CEDAR) University of Cambridge School of Clinical Medicine Cambridge UK; ^3^ Department of Public Health and Primary Care, Strangeways Research Laboratory University of Cambridge Cambridge UK

**Keywords:** Alcohol consumption, drinking, life course, longitudinal study, misclassification error, trajectories

## Abstract

**Background and aims:**

Studies that report the relationship between alcohol consumption and disease risk have predominantly operationalized drinking according to a single baseline measure. The resulting assumption of longitudinal stability may be simplistic and complicate interpretation of risk estimates. This study aims to describe changes to the volume of consumption during the adult life‐course according to baseline categories of drinking.

**Design:**

A prospective observational study.

**Setting:**

United Kingdom.

**Participants:**

A cohort of British civil servants totalling 6838 men and 3372 women aged 34–55 years at baseline, followed for a mean 19.1 (standard deviation = 9.5) years.

**Measurements:**

The volume of weekly alcohol consumption was estimated from data concerning the frequency and number of drinks consumed. Baseline categories were defined: non‐current drinkers, infrequent drinkers, 0.1–50.0 g/week, 50.1–100.0 g/week, 100.1–150.0 g/week, 150.1–250.0 g/week and >250.0 g/week. For women, the highest category was defined as > 100.0 g/week. Baseline frequency was derived as ‘daily or almost daily’ and ‘not daily or almost daily’. Trajectories were estimated within baseline categories using growth curve models.

**Findings:**

Trajectories differed between men and women, but were relatively stable within light‐to‐moderate categories of baseline consumption. Drinking was least stable within the highest categories of baseline consumption (men: > 250.0 g/week; women: > 100.0 g/week), declining by 47.0 [95% confidence interval (CI) = 40.7, 53.2] and 16.8 g/week (95% CI = 12.6, 21.0), respectively, per 10‐year increase in age. These declines were not a consequence of sudden transitions to complete abstention. Rates of decline appear greatest in older age, with trajectories converging toward moderate volumes.

**Conclusion:**

Among UK civil servants, consumption within baseline drinking categories is generally stable during the life‐course, except among heavier baseline drinkers, for whom intakes decline with increasing age. This shift does not appear to be driven by transitions to non‐drinking. Cohorts of older people may be at particular risk of misclassifying former heavy drinkers as moderate consumers of alcohol.

## Introduction

An extensive body of research has explored the dose–response association between alcohol consumption and assorted negative health events 1, 2, 3, 4. However, the majority of existing studies have operationalized drinking according to a single baseline measure of self‐reported alcohol consumption. For instance, of the 38 longitudinal studies analysed as part of a recent meta‐analysis into the effect of drinking upon the risk of Type 2 diabetes 2, only one had utilized data from subsequent phases of follow‐up 5. In so doing, constituent studies assume that drinking is stable during the follow‐up period, but there is reason to doubt this. As detailed elsewhere 6, 7, alcohol consumption appears to vary markedly as a function of age, with disparate trajectories reported throughout the adult life‐course. As noted by a meta‐analysis of alcohol consumption and cardiovascular disease 1, the cross‐sectional categorization of participants into drinking categories risks provides a poor operationalization of consumption during the life‐course, particularly in studies of longer duration.

While the limitations of single alcohol measures have been discussed within the literature, much of the focus has been directed towards the need to disaggregate heterogeneous non‐drinkers 8, 9 and infrequent drinkers 9 reliably, owing to their disparate risks of assorted health conditions 4, 10. Such discussions overlook the risk of misclassification error among current drinkers whose alcohol consumption changes as a function of age 7. At least three studies have reported the longitudinal stability of intake within baseline‐defined categories of drinking, with each having pooled heterogeneous groups of infrequent and non‐drinkers and modelled changes as a function of follow‐up time 11, 12, 13. An understanding of how alcohol consumption varies within a broad spectrum of disparate drinking groups is therefore limited, especially within a life‐course context.

To elaborate upon the issue, this study aimed to: (a) quantify the stability of drinking across the adult life‐course according to baseline categories of consumption; and (b) establish the presence of a sex interaction, given sex‐specific differences in the mean trajectory of consumption throughout the adult life course 7. The study also includes two *post‐hoc* analyses. The first reports within‐category differences in the trajectory of alcohol consumption according to the frequency of baseline consumption, owing to a greater regularity of drinking with increasing age 14 and the possibility that this may be associated with the volume of consumption. The second describes changes to the probability of transition from drinking to non‐drinking throughout the adult life‐course. This final analysis tackles a limitation of the primary analyses, which estimate mean drinking trajectories and so provide no indication as to how participants transition between drinking categories with increasing age. Such analyses help to reveal whether declining trajectories occur as a consequence of a general decrease in consumption, or a sudden transition among some constituent drinkers to complete abstention.

## Methods

### Design

The Whitehall II cohort was established in 1985 and enlisted 10 308 (6895 male and 3413 female) civil servants aged 34–55 years who worked in the offices of 20 Whitehall departments 15. Initial measurements were obtained between 1985 and 1988 via a self‐administered questionnaire and clinical examination. Participants were then followed‐up at regular intervals to produce 11 phases of data by 2012–13. The University College London Medical School Committee on the ethics of human research approved the Whitehall II study. Whitehall II data are available to bona fide researchers for research purposes. Please refer to the Whitehall II data sharing policy at http://www.ucl.ac.uk/whitehallII/datasharing.

### Measures

Drinking data were extracted from all phases at which alcohol consumption questions were incorporated: phases 1 (1985–88), 2 (1989–90), 3 (1991–93), 5 (1997–99), 7 (2003–04), 9 (2007–09) and 11 (2012–13). Participants were asked to report the frequency with which they consumed alcohol during the year preceding interview. This information was used to derive a baseline frequency variable: ‘daily or almost daily’ and ‘not daily or almost daily’.

Those who reported drinking alcohol during the preceding year were then asked to declare the number of alcoholic drinks they had consumed during the week prior to interview according to ‘measures’ of spirits, ‘glasses’ of wine or ‘pints’ of beer or cider. A conservative 8 g of alcohol is assumed per measure of spirits or glass of wine, and 16 g for each pint of beer or cider. Measurements were aggregated to derive the total grams of alcohol consumed during the week prior to interview among current drinkers.

Baseline consumption categories were defined using volume and frequency data reported at phase 1: non‐current drinkers (no alcohol consumption throughout the year prior to interview), infrequent drinkers (consumed alcohol in the year preceding interview but did not drink in the week prior to measurement), 0.1–50.0 g/week, 50.1–100.0 g/week, 100.1–150.0 g/week, 150.1–250.0 g/week and > 250.0 g/week. For women, among whom the volume of alcohol consumption was lower, the top drinking categories were merged (> 100.0 g/week).

### Statistical analysis

#### Primary analyses

Age in years was selected as the time‐scale, scaled to the minimum age at baseline (34.1 years). Sensitivity analyses are also reported that used follow‐up in years as the time‐scale.

The linear mean trajectory of alcohol consumption was estimated for each baseline drinking category using linear growth curve models via the ‐mixed‐ command in Stata version 13 16. To determine whether the longitudinal trajectories differed significantly between men and women, a three‐way interaction was modelled in the first instance between sex, baseline consumption category and age.

Owing to the degree of variability in alcohol consumption present within and between individuals, both random intercepts and random slopes were permitted and Anniecovariance between repeated measures allowed to take any form. Additionally, given that alcohol consumption was positively skewed, robust standard errors were calculated to avoid applying a transformation and thereby aid interpretability.

Non‐linear trajectories were explored by subjecting age to a restricted range of fractional polynomial transformations (x^−2^, x^−1^, x^–0.5^, ln(x), x^0.5^, x^1^, x^2^ and x^3^), which permit the modelling of monotonic and non‐monotonic relationships between alcohol consumption and age 17. The fit of each transformation was assessed according to the Bayesian information criterion (BIC) 18. Owing to a lack of convergence when some non‐linear transformations were applied, BICs were calculated for simplified models that constrained to zero any covariance between repeated measures. An improvement in fit relative to a linear model was defined as any reduction in the BIC greater than or equal to a value of 10, which is described as a strong indicator of an improvement to model specification 19. The best‐fitting trajectory for each baseline category was then plotted allowing random effects and an unstructured covariance matrix, as per the primary linear models.

Given the risk of selection bias in circumstances where underlying missingness mechanisms are informative, a chained equations imputation model was created under the assumption that missing drinking data were predictable from observed covariates 20, 21. Using the ‐mi‐ package 22, missing data for participants who were lost to follow‐up or else provided no response to the alcohol consumption questions of interest were predicted from a range of demographic, socio‐economic, health and life‐style characteristics. A total of 50 imputations were run to be sure of capturing appropriately the degree of uncertainty surrounding the predicted values, with iterations run for each imputation until predicted values reached convergence. The imputation model excluded missing data for phases of observation on or after any documented date of death. Finally, to ensure that the estimation sample was consistent between imputations, baseline consumption categories were defined using the observed data only, with imputed volumes of alcohol consumption then predicted for all follow‐up phases.

#### Post‐hoc analyses

The first *post‐hoc* analysis restricted the primary linear models to current drinkers, then included a three‐way interaction between age, baseline consumption category and baseline consumption frequency.

For the second *post‐hoc* analysis, logit models were constructed to estimate the probability of transition to non‐drinking during the life‐course within each baseline category of current drinkers. Sex‐specific binary variables were coded for each such category according to whether or not constituent participants had transitioned to non‐drinking at a given phase of observation. The ‐xtlogit‐ command was used to predict the probability of transition to non‐drinking within each baseline consumption category, with the predicted probabilities then plotted as a function of age 23.

## Results

### Descriptive statistics

Of the 72 156 potential person‐observations captured over seven phases of follow‐up, 4.8% (*n* = 3432) were missing due to mortality and 18.8% (*n* = 13 563) were lost to follow‐up. Of the 55 161 valid person‐observations, 0.9% (*n* = 481) were missing due to item non‐response at baseline and 3.3% (*n* = 1823) missing due to missing volume data between phases.

The weekly volume of alcohol consumption was thus measured from baseline throughout 36 349 person‐observations among men and 16 208 person‐observations among women, as reported by 6838 and 3372 participants, respectively. Participants were aged 34.1–56.3 years at baseline and followed for a mean 19.1 [standard deviation (SD) = 9.5] years, capturing consumption during a period of the adult life course ranging from 34.1 to 83.6 years of age. Relative to categories of current drinkers, baseline non‐drinkers were more likely to be of non‐white ethnic background, in fair or poor health, low occupational grade, physically active or current smokers (Supporting information, Appendix S1).

### Linear growth curve models

A three‐way interaction between sex, baseline consumption category and age revealed differences between men and women in both the volume of consumption within each category at baseline (*P* < 0.001) and the category‐specific rates of change with increasing age (*P* < 0.001). Accordingly, sex‐specific results are reported hereafter.

**Figure 1:**
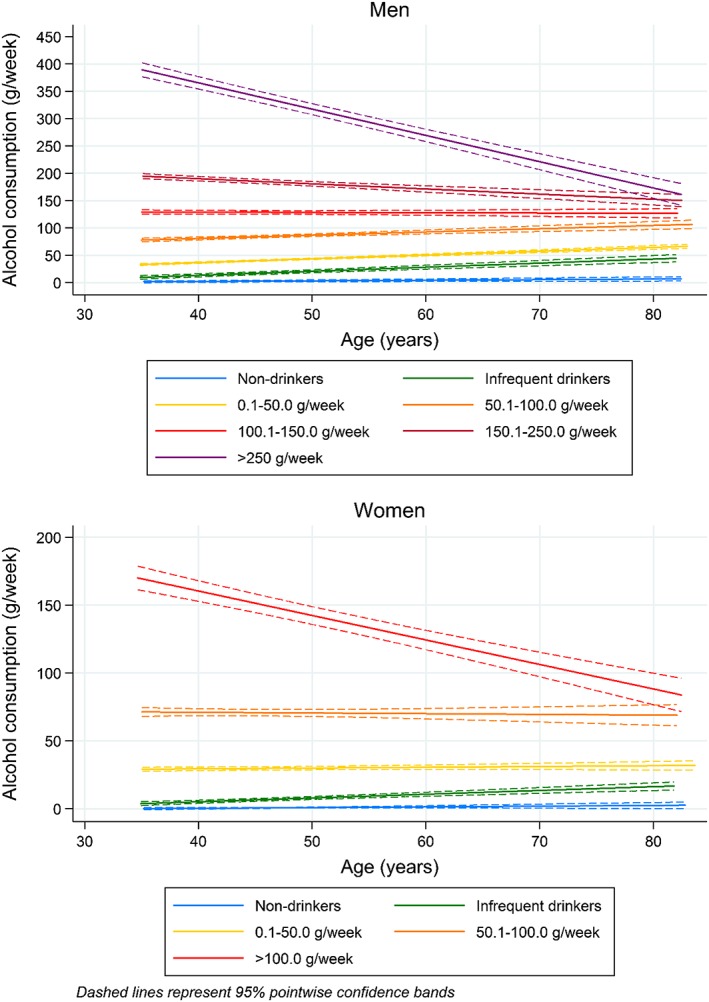
Linear trajectories of mean weekly volume of alcohol consumption between the ages of 34–84 years, stratified by sex and baseline alcohol consumption category

Among men and women, a total 12.2 and 10.0% of variability in alcohol intake was explained by within‐subject changes with increasing age. Consumption changed within a number of baseline consumption categories during the adult life‐course, with slopes appearing to converge towards moderate levels (Table 1, Fig. 1).

**Table 1:** Mean weekly volume of alcohol consumption according to a two‐way interaction between the baseline category of alcohol consumption and age, stratified by sex.

Linear growth curve models	Sample (n)	Mean g/week (95% CI)	P‐value
**Men**			
Consumption volume			
Intercept		1.4 (−0.3, 3.1)	0.110
Change per 10‐year increase in age		1.1 (0.3, 2.0)	0.010
Difference in baseline consumption			
Non‐drinker	220	Reference	
Infrequent drinker	669	6.8 (3.0, 10.6)	< 0.001
0.1–50.0 g/week	2073	30.3 (27.9, 32.7)	< 0.001
50.1–100.0 g/week	1432	75.1 (71.4, 78.9)	< 0.001
100.1–150.0 g/week	881	127.7 (123.4, 132.0)	< 0.001
150.1–250.0 g/week	915	194.7 (189.5, 199.8)	< 0.001
>250.0 g/week	648	389.3 (376.0, 402.5)	< 0.001
Difference in the decennial rate of change			
Non‐drinker		Reference	
Infrequent drinker		6.6 (4.6, 8.6)	< 0.001
0.1–50.0 g/week		6.0 (4.8, 7.3)	< 0.001
50.1–100.0 g/week		5.0 (2.8, 7.2)	< 0.001
100.1–150.0 g/week		−1.6 (−3.9, 0.7)	0.177
150.1–250.0 g/week		−10.7 (−13.6, −7.8)	< 0.001
>250.0 g/week		−48.1 (−53.5, −42.7)	< 0.001
**Women**			
Consumption volume			
Intercept		−0.1 (−1.2, 1.0)	0.810
Change per 10‐year increase in age		0.6 (−0.2, 1.3)	0.150
Difference in baseline consumption			
Non‐drinker	216	Reference	
Infrequent drinker	764	2.5 (0.7, 4.3)	0.006
0.1–50.0 g/week	1428	28.7 (26.8, 30.5)	< 0.001
50.1–100.0 g/week	542	71.4 (67.9, 74.9)	< 0.001
> 100.0 g/week	422	167.9 (159.2, 176.5)	< 0.001
Difference in the decennial rate of change			
Non‐drinker		Reference	
Infrequent drinker		2.6 (1.5, 3.7)	< 0.001
0.1–50.0 g/week		0.2 (−1.0, 1.3)	0.771
50.1–100.0 g/week		−1.0 (−3.2, 1.1)	0.356
> 100.0 g/week		−17.4 (−20.8, −13.9)	< 0.001

CI = confidence interval.

The magnitude of these changes was most pronounced among participants within the highest baseline categories (men: > 250.0 g/week; women: > 100.0 g/week), where the volume of consumption fell by an average 47.0 g/week [95% confidence interval (CI) = 40.7, 53.2] among men and 16.8 g/week (95% CI = 12.6, 21.0) among women per 10‐year increase in age (each calculated as the coefficient for the average rate of change per decade increase in age within the referent category, plus the group‐specific change per decade increase in age). Changes within most other categories were comparatively small, indicating that light and moderate categories of baseline alcohol consumption were largely stable during the period of the life‐course captured by the Whitehall II study. Longitudinal trends between baseline categories were comparable when follow‐up time was adopted as the time‐scale, with adjustment for date of birth (Supporting information, Appendix S2).

Results based upon the imputed data set are reported in the Supporting information, Appendix S3. Relative to the complete‐case model (Table 1), baseline volumes of alcohol consumption were slightly higher within each drinking category, with rates of change shifted consistently towards the negative.

### Non‐linear growth curve models

**Figure 2:**
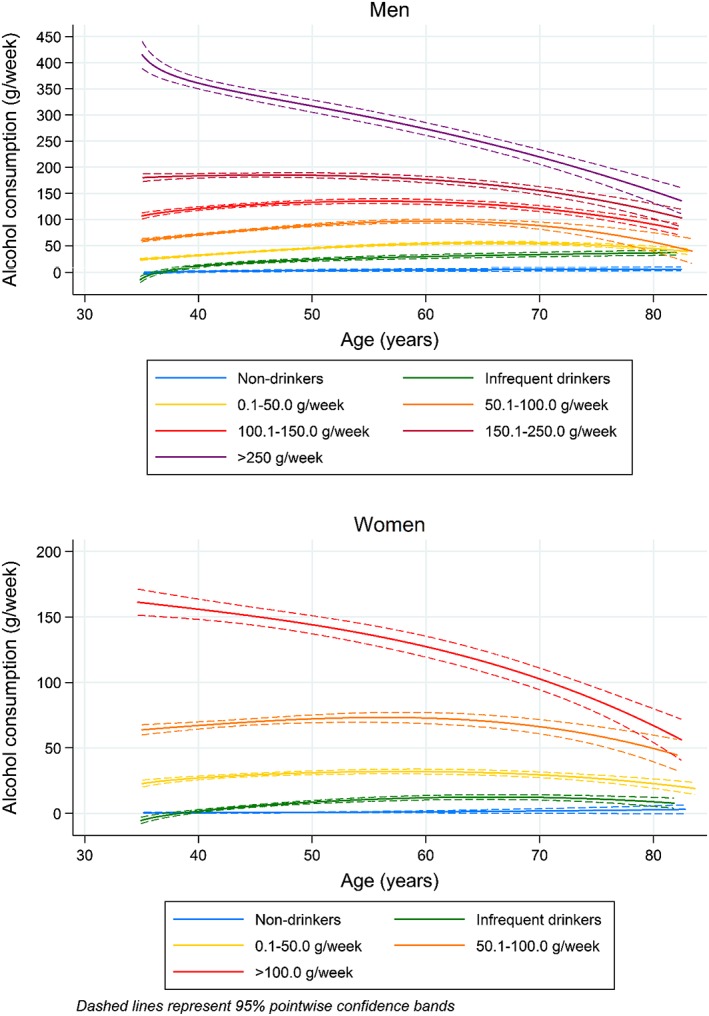
Non‐linear trajectories of mean weekly volume of alcohol consumption between the ages of 34–84 years, stratified by sex and baseline alcohol consumption category

Non‐linear slopes provided an improvement in fit for all but male and female baseline non‐drinkers, for whom drinking remained stable with age. In addition to the gradual convergence towards moderate volumes of consumption evident in Fig. 1, the non‐linear trajectories show that consumption within all baseline categories of current drinking declined from approximately 60 to 65 years of age onwards (Fig. 2).

### Consumption frequency

Supporting information, Appendix S4 reports results from analyses that included a three‐way interaction between age, baseline category of drinking volume and baseline consumption frequency. Despite within‐category differences in the volume of consumption at baseline according to whether or not participants reported drinking on a ‘daily’ or ‘almost daily’ basis, there was no difference in the rate of change across the adult life‐course by frequency.

### Transitions to non‐drinking

**Figure 3:**
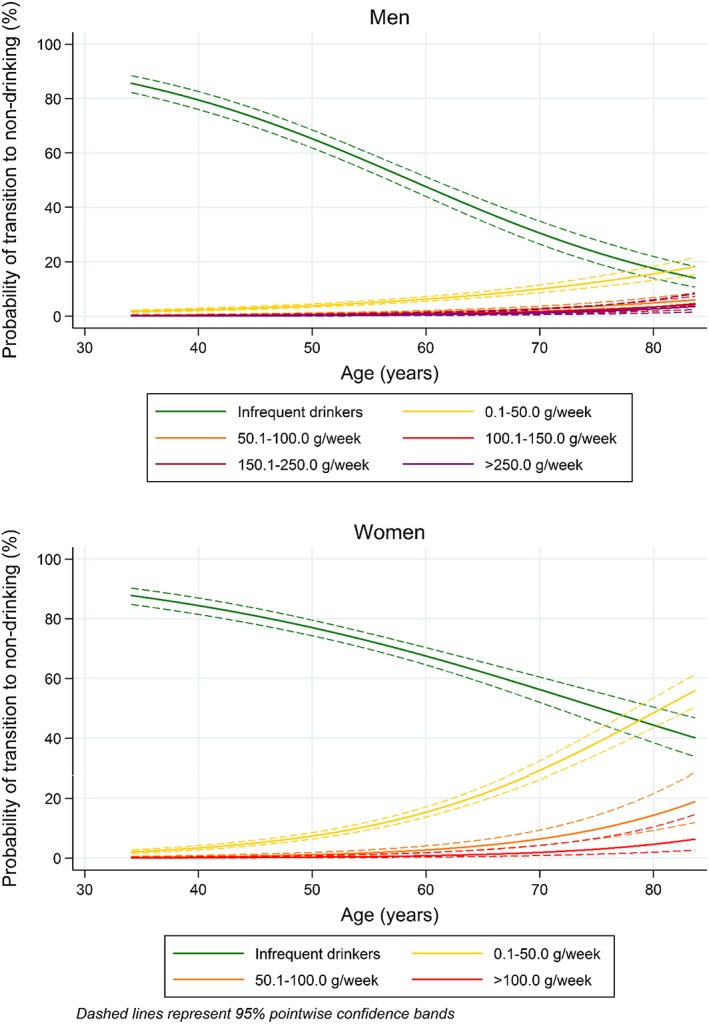
Probability of transition to non‐drinking across the adult life–course, stratified by baseline alcohol consumption category

As shown in Fig. 3, the likelihood of transition to abstention increased as a function of age among all baseline categories except infrequent drinkers, indicating that current drinkers were most likely to stop drinking in older age. Interestingly, despite participants within the heaviest baseline drinking categories (men: > 250.0 g/week; women: > 100.0 g/week) exhibiting the greatest rates of decline in mean consumption with increasing age (Fig. 1), the probability of transition to non‐drinking remained consistently low across the adult life‐course.

## Discussion

This study investigated the stability of alcohol consumption categories throughout the adult life‐course, when defined according to a single measure recorded at baseline. During a period of 50 years, intakes within baseline‐defined consumption categories were found to vary in a manner concordant with results from at least two other studies, whereby less longitudinal stability present within higher categories of baseline consumption as a function of follow‐up time 12, 13. This was in contrast to a study of 5‐year changes to alcohol consumption among post‐menopausal women, where the proportion of transitions between categories of current drinkers were roughly equivalent regardless of the volume consumed at baseline 12.

Results from the *post‐hoc* logit models indicate that downward trajectories observed among heavier baseline drinkers (men: > 150.0 g/week; women: > 100.0 g/week) were unlikely to have been a consequence of sudden transitions to complete abstention, but of a general reduction in drinking with increasing age. Reasons for this attenuation are likely to be complex, including a response to declining health or a proactive health precaution 24, 25. This longitudinal convergence of drinking trajectories with increasing age suggests that the categorization of drinkers using a single baseline measure may be especially problematic when applied to cohorts of older populations. Specifically, with higher volumes of alcohol consumption associated elsewhere with an increased risk of adverse health conditions 26, 27, the misclassification of former heavy drinkers as moderate consumers may lead to an overestimation of risk among older moderate drinkers. This convergence may explain why reductions in the risk of coronary heart disease 28 or all‐cause mortality 29 at moderate volumes of consumption appear less pronounced within adults who were older at baseline.

The tendency of observational studies to model drinking according to only a single measure of exposure thus ignores changes to alcohol intake throughout the life‐course and the possible effect of such variation on disease risk. For example, in an analysis of Type 2 diabetes risk, a significant interaction is reported between the volume of consumption at baseline and changes to exposure over time 13, whereby reductions in risk are apparent only among moderate drinkers (< 15 g/day) who increased their consumption over time. A similar finding has been reported for coronary heart disease 30. Elsewhere, study participants who drank heavily during early adulthood exhibit a greater risk of metabolic syndrome and common cardiovascular risk factors relative to participants with stable trajectories of consumption 31, with less stable drinking trajectories having been associated with a higher risk of mortality irrespective of average consumption 32.

Such papers illustrate how the direction and timing of longitudinal changes to drinking behaviour throughout the adult life‐course may represent important modifiers of disease risk that are largely overlooked by contemporary research. Although the number and frequency of repeated measures reported by existing studies are variable, and no study is yet to capture drinking behaviours throughout the whole adult life‐course 7, 33, the use of repeated measures is important if differences in risk between heterogeneous consumption trajectories are to be understood more clearly and sensitive periods identified during which particular alcohol consumption behaviours may be most harmful, aiding the targeting of alcohol reduction interventions 34. To date, however, there is no consistent approach to handling such data, with some opting to restrict analyses to participant whose consumption was stable within pre‐defined limits 35, or else categorizing participants according to whether their drinking increased, decreased or remained stable over a given period of time 13. In addition, there is no clear agreement concerning the appropriate means of classifying drinkers who cease consumption prior to baseline measurement. A number of proposals have been put forward, including an intention‐to‐treat approach, which assigns former drinkers with a current drinking value predicted to be most representative of their prior consumption 36, or the use of a retrospective life grid as a means of soliciting participants to estimate their past consumption 37. Although the best approach for dealing with both issues will differ to some extent according to the aim of the study and the data available, the incorporation and treatment of longitudinal data for the analysis of alcohol‐related risks represents an important area for future debate.

### Strengths and limitations

Although other studies have reported the stability of consumption according to baseline categories of drinking 11, 12, 13, this is the first study of which we are aware to describe changes from a life‐course perspective as opposed to shifts during follow‐up. Analyses benefited from seven phases of observation covering almost 50 years of the adult life‐course. Although representing a geographically concentrated and occupationally narrow cohort, trajectories derived from Whitehall II data are consistent with those reported from nationally representative, UK‐based cohorts 7. This increases our confidence that trajectories stratified by baseline consumption should be generalizable to other cohorts.

Despite these benefits, the data set lacks prospective alcohol consumption data during early adulthood and advanced old age. Based on existing research 7, it is hypothesized that the latter is probably marked by a continued convergence towards lower volumes of consumption. This is intimated by the non‐linear trajectories presented in Fig. 2. As such, longitudinal transitions between baseline categories may be even more pronounced during periods of the life‐course not captured by the Whitehall II study. However, while correlations between intercepts and rates of change were negative, there is a possibility that the convergence of drinking trajectories was a result of regression to the mean and not a consequence of age‐related factors.

Analyses were dependent upon self‐reported measures of alcohol consumption and so potentially subject to a degree of inaccuracy owing to reporting and recall biases 38 and measurement error 39. Furthermore, current drinking was derived from questions that concern consumption during the week prior to interview. It is possible that these provide a poor surrogate for true average weekly consumption, with quantity–frequency questionnaires tending to produce lower drinking estimates than graduated frequency questionnaires 38, which may be more effective at capturing episodic heavy drinking 40. Drinking diaries also show promise as a more accurate means of estimating consumption 41. Regardless, observed issues of longitudinal stability will apply to data obtained using any means of self‐reported questionnaire, with implications for all studies of alcohol, whether cross‐sectional or longitudinal in design. Finally, reported analyses show just one of many different dimensions of drinking behaviour, such as differences by drink type and episodic heavy consumption.

In summary, baseline‐defined categories of alcohol consumption appear largely stable across the life‐course among both sexes, except for heavier drinkers, where intake declined markedly with increasing age. These downward trajectories do not seem to be driven by transitions to non‐drinking, indicating that attenuations to the volume of consumption may be gradual. Owing to unstable trajectories among heavier baseline drinkers, there is an indication that cohorts of older people are at particular risk of misclassifying former heavy drinkers as moderate consumers of alcohol. This may have implications for risk estimates derived in studies of predominantly older adults.

### Ethics approval

The University College London Medical School Committee on the ethics of human research approved the Whitehall II study. Whitehall II data are available to bona fide researchers for research purposes. Please refer to the Whitehall II data sharing policy at http://www.ucl.ac.uk/whitehallII/datasharing.

## Declaration of interests

None.

## Supporting information


**Appendix S1** Descriptive statistics as reported at baseline.
**Appendix S2** Linear trajectories of mean weekly volume of alcohol consumption over the period of follow‐up, adjusted for date of birth and stratified by sex and baseline consumption category.
**Appendix S3** Mean weekly volume of alcohol consumption according to a linear two‐way interaction between the baseline category of alcohol consumption and age, stratified by sex. Multiply imputed data.
**Appendix S4** The mean weekly volume of alcohol consumption according to a linear three‐way interaction between the baseline category of alcohol consumption, baseline frequency of alcohol consumption and age, stratified by sex.
