# Supplementary material for: The stability of baseline‐defined categories of alcohol consumption during the adult life‐course: a 28‐year prospective cohort study
Source: Addiction. 2017 Aug 24;113(1):34–43. doi: 10.1111/add.13949 (PMC5725237; doi:10.1111/add.13949)
Supplement: Supplementary file 1 — Appendix S1 Descriptive statistics as reported at baseline. Appendix S2 Linear trajectories of mean weekly volume of alcohol consumption over the period of follow‐up, adjusted for date of birth and stratified by sex and baseline consumption category. Appendix S3 Mean weekly volume of alcohol consumption according to a linear two‐way interaction between the baseline category of alcohol consumption and age, stratified by sex. Multiply imputed data. Appendix S4 The mean weekly volume of alcohol consumption according to a linear three‐way interaction between the baseline category of alcohol consumption, baseline frequency of alcohol consumption and age, stratified by sex. [file ADD-113-34-s001.docx]

**Supporting information**

**Appendix 1 Descriptive statistics as reported at baseline**

|  | **Non-drinkers** | |  | **Infrequent drinkers** | | |  | | **0.1-50.0g/week** | |  | **50.1-100.0g/week** | |  | **>100.0g/week** | | |
| --- | --- | --- | --- | --- | --- | --- | --- | --- | --- | --- | --- | --- | --- | --- | --- | --- | --- |
| **Variable** | **% (95% CI)** | **n** |  | **% (95% CI)** | **n** | |  | | **% (95% CI)** | **n** |  | **% (95% CI)** | **n** |  | **% (95% CI)** | **n** | |
| **Age** |  |  |  |  |  | |  | |  |  |  |  |  |  |  |  | |
| Mean (years) | 45.87 (45.29, 46.46) | 436 |  | 45.89 (45.58, 46.20) | 1,433 | |  | | 45.46 (45.26, 45.66) | 3,501 |  | 44.47 (44.21, 44.74) | 1,974 |  | 43.98 (43.77, 44.20) | 2,866 | |
|  |  |  |  |  |  | |  | |  |  |  |  |  |  |  |  | |
| **Body mass index** |  |  |  |  |  | |  | |  |  |  |  |  |  |  |  | |
| Mean (kg/m^2^) | 24.97 (24.58, 25.36) | 436 |  | 24.93 (24.71, 25.14) | 1,431 | |  | | 24.54 (24.42, 24.67) | 3,497 |  | 24.35 (24.21, 24.50) | 1,971 |  | 24.76 (24.65, 24.88) | 2,861 | |
|  |  |  |  |  |  | |  | |  |  |  |  |  |  |  |  | |
| **Ethnicity^a^** |  |  |  |  |  | |  | |  |  |  |  |  |  |  |  | |
| White | 53.4 (48.6, 58.1) | 229 |  | 82.6 (80.5, 84.4) | 1,169 | |  | | 89.4 (88.3, 90.4) | 3,100 |  | 95.3 (94.3, 96.2) | 1,871 |  | 96.0 (95.2, 96.7) | 2,732 | |
| South Asian | 33.6 (29.2, 38.2) | 144 |  | 10.0 (8.5, 11.6) | 141 | |  | | 5.0 (4.3, 5.8) | 174 |  | 2.6 (2.0, 3.5) | 52 |  | 2.6 (2.0, 3.2) | 73 | |
| Other | 13.1 (10.2, 16.6) | 56 |  | 7.5 (6.2, 9.0) | 106 | |  | | 5.6 (4.9, 6.4) | 194 |  | 2.0 (1.5, 2.8) | 40 |  | 1.4 (1.1, 2.0) | 41 | |
|  |  |  |  |  |  | |  | |  |  |  |  |  |  |  |  | |
| **General health** |  |  |  |  |  | |  | |  |  |  |  |  |  |  |  | |
| Excellent/very good | 62.3 (57.6, 66.8) | 271 |  | 64.1 (61.6, 66.6) | 917 | |  | | 71.6 (70.0, 73.0) | 2,496 |  | 77.4 (75.5, 79.2) | 1,520 |  | 77.9 (76.3, 79.4) | 2,227 | |
| Good | 28.7 (24.7, 33.2) | 125 |  | 28.7 (26.5, 31.1) | 411 | |  | | 23.0 (21.6, 24.4) | 801 |  | 18.8 (17.1, 20.6) | 369 |  | 18.4 (17.0, 19.8) | 525 | |
| Fair/poor | 9.0 (6.6, 12.1) | 39 |  | 7.1 (5.9, 8.6) | 102 | |  | | 5.5 (4.8, 6.3) | 191 |  | 3.8 (3.1, 4.8) | 75 |  | 3.7 (3.1, 4.5) | 107 | |
|  |  |  |  |  |  | |  | |  |  |  |  |  |  |  |  | |
| **Marital status** |  |  |  |  |  | |  | |  |  |  |  |  |  |  |  | |
| Married or cohabiting | 71.0 (66.5, 75.1) | 308 |  | 67.1 (64.7, 69.5) | 960 | |  | | 73.7 (72.2, 75.2) | 2,568 |  | 78.0 (76.1, 79.8) | 1,535 |  | 75.9 (74.3, 77.4) | 2,169 | |
| Single | 19.8 (16.3, 23.9) | 86 |  | 20.8 (18.8, 23.0) | 298 | |  | | 16.8 (15.6, 18.1) | 586 |  | 14.0 (12.5, 15.6) | 275 |  | 15.0 (13.7, 16.4) | 429 | |
| Divorced | 7.6 (5.4, 10.5) | 33 |  | 9.5 (8.1, 11.1) | 136 | |  | | 7.9 (7.0, 8.8) | 275 |  | 7.1 (6.1, 8.3) | 140 |  | 8.3 (7.4, 9.4) | 238 | |
| Widowed | 1.6 (0.8, 3.4) | 7 |  | 2.5 (1.8, 3.5) | 36 | |  | | 1.6 (1.2, 2.0) | 54 |  | 0.9 (0.6, 1.4) | 18 |  | 0.8 (0.5, 1.2) | 23 | |
|  |  |  |  |  |  | |  | |  |  |  |  |  |  |  |  | |
| **Occupational grade^b^** |  |  |  |  |  | |  | |  |  |  |  |  |  |  |  | |
| High | 13.3 (10.4, 16.8) | 58 |  | 12.2 (10.6, 14.0) | 175 | |  | | 26.1 (24.6, 27.6) | 913 |  | 36.1 (34.0, 38.2) | 712 |  | 40.0 (38.2, 41.8) | 1,147 | |
| Middle | 38.1 (33.6, 42.7) | 166 |  | 43.5 (40.9, 46.1) | 623 | |  | | 47.7 (46.0, 49.3) | 1,669 |  | 49.2 (47.0, 51.4) | 971 |  | 51.3 (49.5, 53.1) | 1,470 | |
| Low | 48.6 (43.9, 53.3) | 212 |  | 44.3 (41.8, 46.9) | 635 | |  | | 26.2 (24.8, 27.7) | 919 |  | 14.7 (13.2, 16.4) | 291 |  | 8.7 (7.7, 9.8) | 249 | |
|  |  |  |  |  |  | |  | |  |  |  |  |  |  |  |  | |
| **Physical activity^c^** |  |  |  |  |  | |  | |  |  |  |  |  |  |  |  | |
| Active | 33.1 (28.8, 37.7) | 140 |  | 23.6 (21.4, 25.9) | 328 | |  | | 15.4 (14.2, 16.6) | 531 |  | 10.7 (9.4, 12.2) | 209 |  | 9.7 (8.6, 10.8) | 275 | |
| Moderately active | 31.9 (27.6, 36.5) | 135 |  | 35.3 (32.8, 37.8) | 491 | |  | | 38.8 (37.2, 40.5) | 1,339 |  | 39.7 (37.6, 41.9) | 776 |  | 37.3 (35.5, 39.1) | 1,060 | |
| Inactive | 35.0 (30.6, 39.7) | 148 |  | 41.2 (38.6, 43.8) | 573 | |  | | 45.8 (44.1, 47.4) | 1,578 |  | 49.6 (47.3, 51.8) | 968 |  | 53.1 (51.2, 54.9) | 1,509 | |
|  |  |  |  |  |  | |  | |  |  |  |  |  |  |  |  | |
| **Sex** |  |  |  |  |  | |  | |  |  |  |  |  |  |  |  | |
| Male | 50.5 (45.8, 55.2) | 220 |  | 46.7 (44.1, 49.3) | 669 | |  | | 59.2 (57.6, 60.8) | 2,073 |  | 72.5 (70.5, 74.5) | 1,432 |  | 85.3 (83.9, 86.5) | 2,444 | |
| Female | 49.5 (44.8, 54.2) | 216 |  | 53.3 (50.7, 55.9) | 764 | |  | | 40.8 (39.2, 42.4) | 1,428 |  | 27.5 (25.5, 29.5) | 542 |  | 14.7 (13.5, 16.1) | 422 | |
|  |  |  |  |  |  | |  | |  |  |  |  |  |  |  |  | |
| **Smoking status** |  |  |  |  |  | |  | |  |  |  |  |  |  |  |  | |
| Current smoker | 72.1 (67.6, 76.1) | 312 |  | 54.4 (51.8, 57.0) | 775 | |  | | 56.5 (54.8, 58.1) | 1,964 |  | 48.0 (45.8, 50.3) | 941 |  | 36.1 (34.4, 37.9) | 1,029 | |
| Former smoker | 15.0 (11.9, 18.7) | 65 |  | 24.6 (22.4, 26.9) | 350 | |  | | 27.4 (25.9, 28.9) | 952 |  | 35.4 (33.3, 37.6) | 694 |  | 41.9 (40.1, 43.8) | 1,195 | |
| Never smoker | 12.9 (10.1, 16.5) | 56 |  | 21.0 (19.0, 23.2) | 299 | |  | | 16.2 (15.0, 17.4) | 562 |  | 16.5 (15.0, 18.3) | 324 |  | 21.9 (20.5, 23.5) | 625 | |
| ^a^Other: includes participants of black ethnic background, Chinese origin or mixed race. | | | | | | | | | |  |  |  |  |  |  |  | |
| ^b^High: administrative; Middle: professional or executive; Low: Clerical or support. | | | | | |  | |  | |  |  |  |  |  |  |  | |
| ^c^Active: ≥150 minutes of moderate-intensity exercise per week, or ≥75 minutes of vigorous-intensity activity; Inactive: <60 minutes of moderate physical activity and <60 minutes of vigorous physical activity; Moderately active: not inactive or active. | | | | | | | | | | | | | | | | |  |
| Sample sizes differ according to item non-response at baseline. Differences between alcohol consumption categories were statistically significant (p<0.001) in all instances, as determined using Wald tests. | | | | | | | | | | | | | | | | |  |

**Appendix 2 Linear trajectories of mean weekly volume of alcohol consumption over the period of follow-up, adjusted for date of birth and stratified by sex and baseline consumption category**

**
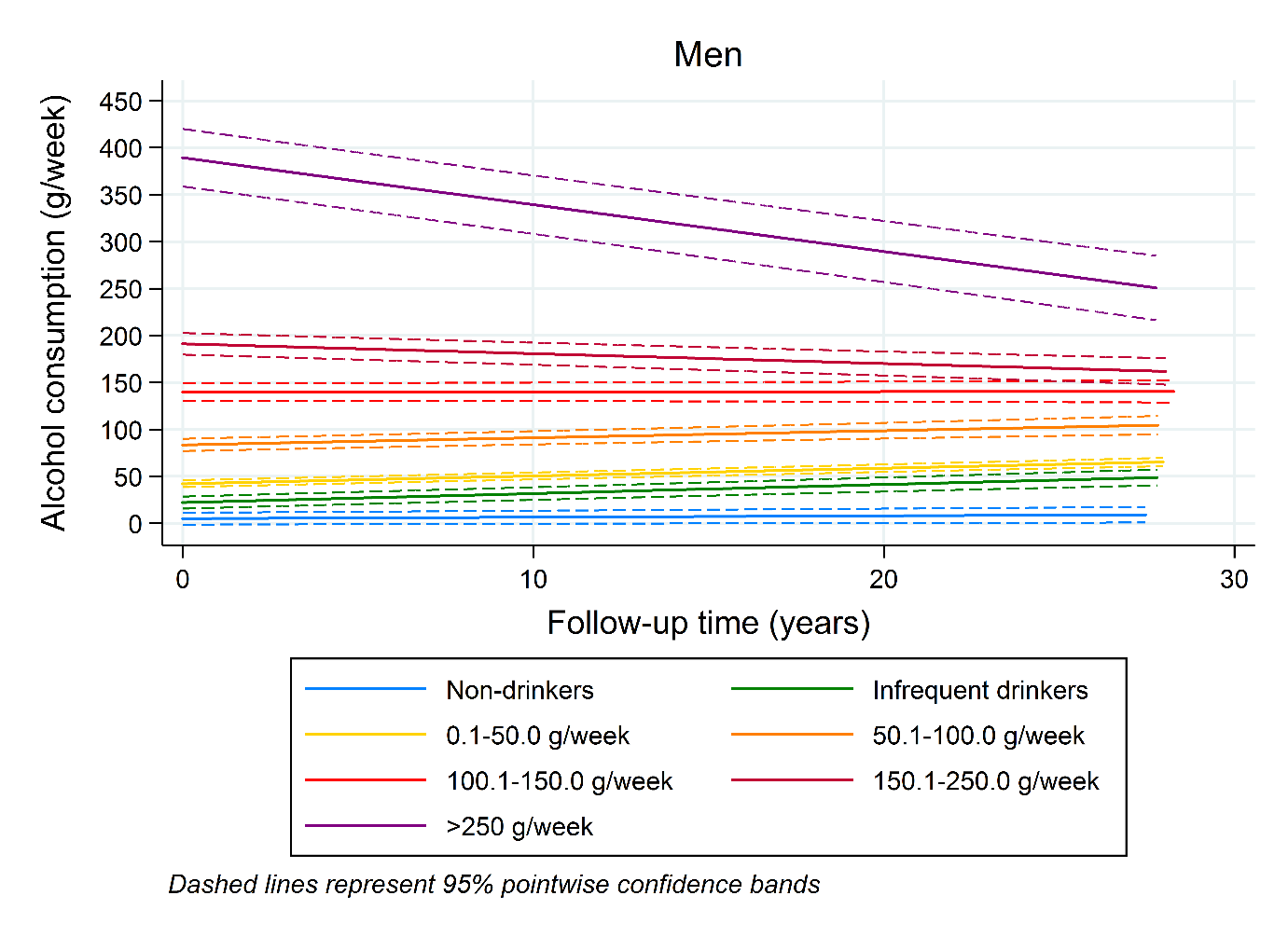

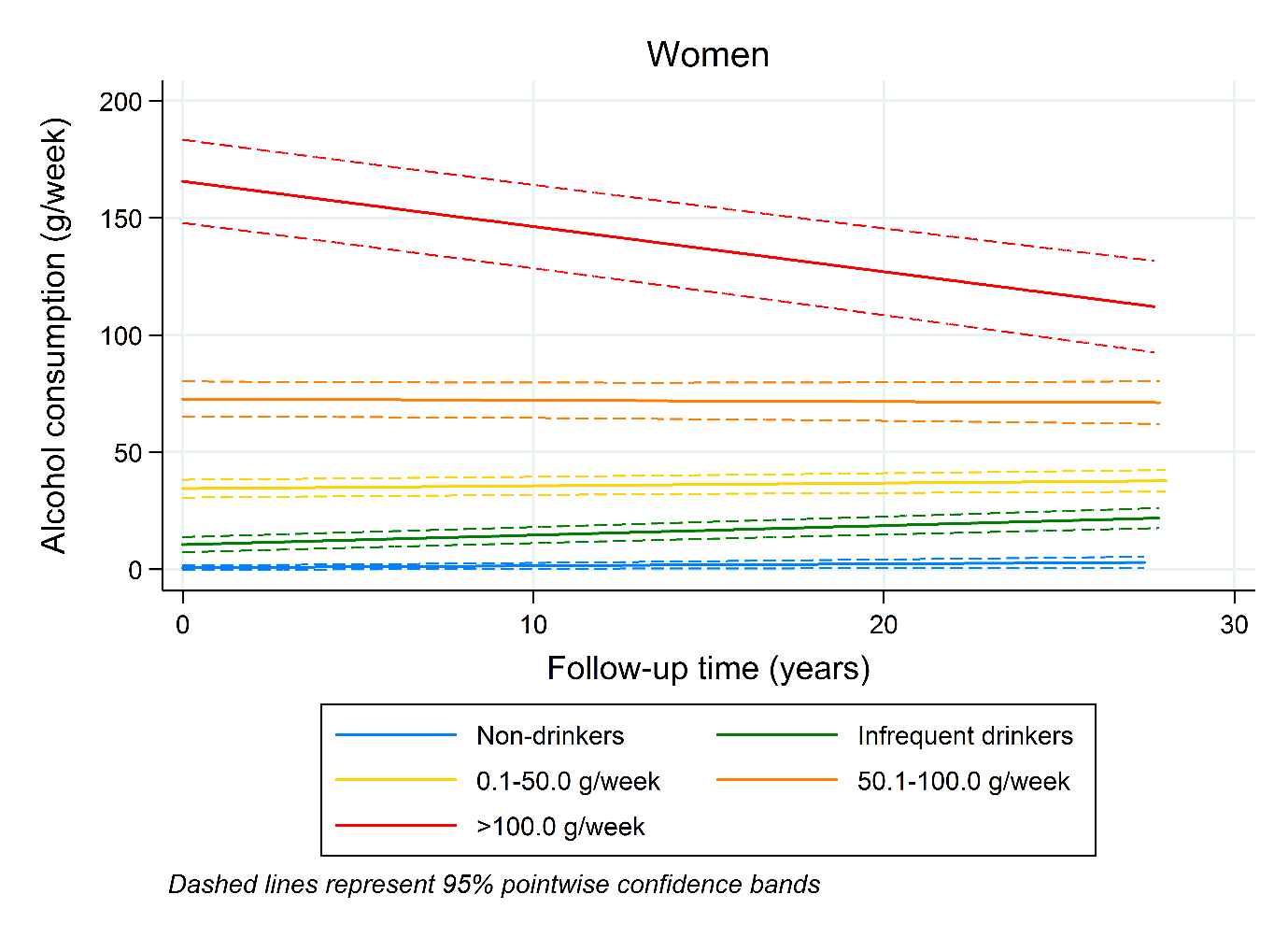
**

**Appendix 3 Mean weekly volume of alcohol consumption according to a linear two-way interaction between the baseline category of alcohol consumption and age, stratified by sex. Multiply imputed data.**

| **Linear growth curve models** | **Sample (n)** | **Mean g/week (95% CI)** | **p-value** |
| --- | --- | --- | --- |
| **Men** |  |  |  |
| **Consumption volume** |  |  |  |
| Intercept |  | 1.4 (-0.3, 3.1) | 0.110 |
| Change per 10-year increase in age |  | 4.8 (3.3, 6.4) | <0.001 |
|  |  |  |  |
| **Difference in baseline consumption** |  |  |  |
| Non-drinker | 220 | Reference |  |
| Infrequent drinker | 669 | 9.9 (5.6, 14.2) | <0.001 |
| 0.1-50.0 g/week | 2,073 | 35.3 (31.9, 38.8) | <0.001 |
| 50.1-100.0 g/week | 1,432 | 81.7 (77.4, 86.0) | <0.001 |
| 100.1-150.0 g/week | 881 | 132.3 (126.7, 138.0) | <0.001 |
| 150.1-250.0 g/week | 915 | 196.4 (189.9, 202.9) | <0.001 |
| >250.0 g/week | 648 | 377.1 (362.9, 391.2) | <0.001 |
|  |  |  |  |
| **Difference in the decennial rate of change** |  |  |  |
| Non-drinker |  | Reference |  |
| Infrequent drinker |  | 3.6 (1.5, 5.7) | 0.001 |
| 0.1-50.0 g/week |  | 3.6 (1.8, 5.4) | <0.001 |
| 50.1-100.0 g/week |  | 1.0 (-1.4, 3.3) | 0.421 |
| 100.1-150.0 g/week |  | -6.0 (-8.8, -3.2) | <0.001 |
| 150.1-250.0 g/week |  | -15.0 (-18.4, -11.7) | <0.001 |
| >250.0 g/week |  | -53.9 (-59.5, -48.3) | <0.001 |
|  |  |  |  |
| **Women** |  |  |  |
| **Consumption volume** |  |  |  |
| Intercept |  | -1.2 (-3.0, 0.7) | 0.207 |
| Change per 10-year increase in age |  | 3.9 (2.8, 5.1) | <0.001 |
|  |  |  |  |
| **Difference in baseline consumption** |  |  |  |
| Non-drinker | 216 | Reference |  |
| Infrequent drinker | 764 | 3.3 (1.0, 5.7) | 0.005 |
| 0.1-50.0 g/week | 1,428 | 31.4 (28.7, 34.2) | <0.001 |
| 50.1-100.0 g/week | 542 | 74.9 (70.4, 79.3) | <0.001 |
| >100.0 g/week | 422 | 166.0 (155.6, 176.3) | <0.001 |
|  |  |  |  |
| **Difference in the decennial rate of change** |  |  |  |
| Non-drinker |  | Reference |  |
| Infrequent drinker |  | 2.1 (0.7, 3.5) | 0.004 |
| 0.1-50.0 g/week |  | -0.5 (-2.0, 1.0) | 0.502 |
| 50.1-100.0 g/week |  | -3.5 (-5.9, -1.1) | 0.005 |
| >100.0 g/week |  | -21.2 (-25.1, -17.2) | <0.001 |

**Appendix 4 The mean weekly volume of alcohol consumption according to a linear three-way interaction between the baseline category of alcohol consumption, baseline frequency of alcohol consumption and age, stratified by sex**

|  |  |  |  |
| --- | --- | --- | --- |
| **Linear growth curve models** | **Sample (n)** | **g/week (95% CI)** | **p-value** |
| **Men** |  |  |  |
| **Consumption volume** |  |  |  |
| Baseline consumption |  | 30.5 (28.8, 32.1) | <0.001 |
| Change to consumption per 10-year increase in age |  | 7.2 (6.2, 8.1) | <0.001 |
| Difference in baseline consumption, by baseline consumption frequency^a^ |  | 27.7 (17.6, 37.7) | <0.001 |
| Difference in change to consumption per 10-year increase in age, by baseline consumption frequency^a^ |  | -0.6 (-5.7, 4.6) | 0.830 |
|  |  |  |  |
| **Difference in baseline consumption between baseline categories** |  |  |  |
| 0.1-50.0 g/week | 2,068 | Reference |  |
| 50.1-100.0 g/week | 1,424 | 42.6 (38.2, 47.1) | <0.001 |
| 100.1-150.0 g/week | 880 | 90.6 (84.5, 96.7) | <0.001 |
| 150.1-250.0 g/week | 913 | 144.7 (136.1, 153.4) | <0.001 |
| >250.0 g/week | 648 | 245.5 (212.7, 278.3) | <0.001 |
|  |  |  |  |
| **Difference in baseline consumption between baseline categories, by baseline consumption frequency^a^** |  |  |  |
| 0.1-50.0 g/week | 2,068 | Reference |  |
| 50.1-100.0 g/week | 1,424 | -14.5 (-26.6, -2.4) | 0.019 |
| 100.1-150.0 g/week | 880 | -12.5 (-25.2, 0.3) | 0.056 |
| 150.1-250.0 g/week | 913 | 0.1 (-14.2, 14.5) | 0.985 |
| >250.0 g/week | 648 | 97.0 (60.1, 133.8) | <0.001 |
|  |  |  |  |
| **Difference in the decennial rate of change between baseline categories** | |  |  |
| 0.1-50.0 g/week | 2,068 | Reference |  |
| 50.1-100.0 g/week | 1,424 | -1.8 (-4.6, 1.0) | 0.207 |
| 100.1-150.0 g/week | 880 | -10.0 (-13.2, -6.8) | <0.001 |
| 150.1-250.0 g/week | 913 | -20.6 (-25.4, -15.9) | <0.001 |
| >250.0 g/week | 648 | -52.9 (-69.0, -36.8) | <0.001 |
|  |  |  |  |
| **Difference in the decennial rate of change between baseline categories, by baseline consumption frequency^a^** |  |  |  |
| 0.1-50.0 g/week | 2,068 | Reference |  |
| 50.1-100.0 g/week | 1,424 | 3.0 (-3.5, 9.4) | 0.370 |
| 100.1-150.0 g/week | 880 | 4.5 (-2.2, 11.2) | 0.186 |
| 150.1-250.0 g/week | 913 | 5.6 (-2.1, 13.3) | 0.152 |
| >250.0 g/week | 648 | -0.9 (-18.6, 16.9) | 0.924 |
|  |  |  |  |
| **Women** |  |  |  |
| **Consumption volume** |  |  |  |
| Baseline consumption |  | 27.6 (26.0, 29.1) | <0.001 |
| Change to consumption per 10-year increase in age |  | 0.2 (-0.6, 1.0) | 0.608 |
| Difference in baseline consumption, by baseline consumption frequency^a^ | | 17.2 (9.4, 25.0) | 0.000 |
| Difference in change to consumption per 10-year increase in age, by baseline consumption frequency^a^ |  | 6.5 (1.5, 11.5) | 0.011 |
|  |  |  |  |
| **Difference in baseline consumption between baseline categories** |  |  |  |
| 0.1-50.0 g/week | 1,425 | Reference |  |
| 50.1-100.0 g/week | 541 | 36.9 (32.6, 41.2) | <0.001 |
| >100.0 g/week | 420 | 102.3 (88.7, 115.8) | <0.001 |
|  |  |  |  |
| **Difference in baseline consumption between baseline categories, by baseline consumption frequency^a^** |  |  |  |
| 0.1-50.0 g/week | 1,425 | Reference |  |
| 50.1-100.0 g/week | 541 | -0.9 (-11.1, 9.3) | 0.860 |
| >100.0 g/week | 420 | 30.4 (11.9, 48.9) | 0.001 |
|  |  |  |  |
| **Difference in the decennial rate of change between baseline categories** | |  |  |
| 0.1-50.0 g/week | 1,425 | Reference |  |
| 50.1-100.0 g/week | 541 | -2.6 (-5.3, 0.1) | 0.060 |
| >100.0 g/week | 420 | -20.5 (-27.1, -13.9) | <0.001 |
|  |  |  |  |
| **Difference in the decennial rate of change between baseline categories, by baseline consumption frequency^a^** |  |  |  |
| 0.1-50.0 g/week | 1,425 | Reference |  |
| 50.1-100.0 g/week | 541 | -1.9 (-8.3, 4.6) | 0.565 |
| >100.0 g/week | 420 | -2.4 (-11.5, 6.7) | 0.605 |
| Baseline consumption frequency coded as a binary variable: 0 less than 'daily' or 'almost daily'; 1 'daily' or 'almost daily'. Reported coefficients are relative to participants who consumed alcohol less than 'daily' or 'almost daily'. | | | |
